# Interpretability of Machine Learning Solutions in Public Healthcare: The CRISP-ML Approach

**DOI:** 10.3389/fdata.2021.660206

**Published:** 2021-05-26

**Authors:** Inna Kolyshkina, Simeon Simoff

**Affiliations:** ^1^Analytikk Consulting, Sydney, NSW, Australia; ^2^School of Computer, Data and Mathematical Sciences, Western Sydney University, Sydney, NSW, Australia; ^3^MARCS Institute for Brain, Behaviour and Development, Western Sydney University, Sydney, NSW, Australia

**Keywords:** machine learning, interpretability, public health, data science methodology, CRISP-ML, necessary level of interpretability, interpretability matrix, cross-industry standard process

## Abstract

Public healthcare has a history of cautious adoption for artificial intelligence (AI) systems. The rapid growth of data collection and linking capabilities combined with the increasing diversity of the data-driven AI techniques, including machine learning (ML), has brought both ubiquitous opportunities for data analytics projects and increased demands for the regulation and accountability of the outcomes of these projects. As a result, the area of interpretability and explainability of ML is gaining significant research momentum. While there has been some progress in the development of ML methods, the methodological side has shown limited progress. This limits the practicality of using ML in the health domain: the issues with explaining the outcomes of ML algorithms to medical practitioners and policy makers in public health has been a recognized obstacle to the broader adoption of data science approaches in this domain. This study builds on the earlier work which introduced CRISP-ML, a methodology that determines the interpretability level required by stakeholders for a successful real-world solution and then helps in achieving it. CRISP-ML was built on the strengths of CRISP-DM, addressing the gaps in handling interpretability. Its application in the Public Healthcare sector follows its successful deployment in a number of recent real-world projects across several industries and fields, including credit risk, insurance, utilities, and sport. This study elaborates on the CRISP-ML methodology on the determination, measurement, and achievement of the necessary level of interpretability of ML solutions in the Public Healthcare sector. It demonstrates how CRISP-ML addressed the problems with data diversity, the unstructured nature of data, and relatively low linkage between diverse data sets in the healthcare domain. The characteristics of the case study, used in the study, are typical for healthcare data, and CRISP-ML managed to deliver on these issues, ensuring the required level of interpretability of the ML solutions discussed in the project. The approach used ensured that interpretability requirements were met, taking into account public healthcare specifics, regulatory requirements, project stakeholders, project objectives, and data characteristics. The study concludes with the three main directions for the development of the presented cross-industry standard process.

## 1. Introduction and Background to the Problem

Contemporary data collection and linking capabilities, combined with the growing diversity of the data-driven artificial intelligence (AI) techniques, including machine learning (ML) techniques, and the broader deployment of these techniques in data science and analytics, have had a profound impact on decision-making across many areas of human endeavors. In this context, public healthcare sets priority requirements toward the robustness, security (Qayyum et al., 2021), and interpretability (Stiglic et al., 2020) of ML solutions. We use the term *solution* to denote the algorithmic decision-making scenarios involving ML and AI algorithms (Davenport and Kalakota, 2019). While the early AI solutions for healthcare, like expert systems, possessed limited explanatory mechanisms (Darlington, 2011), these mechanisms proved to have an important role in clinical decision-making and, hence, made healthcare practitioners, clinicians, health economists, patients, and other stakeholders aware about the need to have such capabilities.

Healthcare domain imposes a broad spectrum of unique challenges to contemporary ML solutions, placing much higher demands with respect to interpretability, comprehensibility, explainability, fidelity, and performance of ML solutions (Ahmad et al., 2018). Among these properties of ML solutions, interpretability is particularly important for human-centric areas like healthcare, where it is crucial for the end users to not only have access to an accurate model but also to trust the validity and accuracy of the model, as well as understand how the model works, what recommendation has been made by the model, and why. These aspects have been emphasized by a number of recent studies, most notably in Caruana et al. (2015) and Holzinger et al. (2017), and summarized in the study by Ahmad et al. (2018).

Healthcare, similar to government and business digital services, manufacturing with its industrial internet of things and creative industries, experienced the much celebrated manifestations of “big data,” “small data,” “rich data,” and the increased impact of ML solutions operating with these data. Consequently, the interpretability of such solutions and the explainability of the impact of the judgements they assist to make or have made and, where needed, the rationale of recommended actions and behavior are becoming essential requirements of contemporary analytics, especially in society-critical domains of health, medical analysis, automation, defense, security, finance, and planning. This shift has been further accentuated by the growing worldwide commitment of governments, industries, and individual organizations to address their endeavors toward the United Nations Sustainable Development Goals^1^ and by the data-dependent scientific and technological challenges faced by the rapid response to the COVID-19 pandemic. The later challenges highlight and reinforce the central role of healthcare, backed by science, technology, lateral thinking, and innovative solutions in societal and economic recovery.

Some state-of-the-art overviews, such as Doshi-Velez and Kim (2017) and Gilpin et al. (2019) related to interpretability, as well as more method-focused papers, like Lipton (2018) and Molnar et al. (2019), tend to use interpretability and explainability interchangeably. They also report that the interpretability of ML solutions and the underlying models is not well-defined. The study related to interpretability is scattered throughout a number of disciplines, such as AI, ML, human-computer interaction (HCI), visualization, cognition, and social sciences (Miller, 2019), to name a few of the areas. In addition, the current research seems to focus on particular categories or techniques instead of addressing the overall concept of interpretability.

Recent systematic review studies, Gilpin et al. (2018) and Mittelstadt et al. (2019), have clarified some differences and relationships between interpretability and explainability in the context of ML and AI. In these domains, interpretability refers to the degree of human interpretability of a given model, including “black box” models (Mittelstadt et al., 2019). Machine interpretability of the outcomes of ML algorithms is treated separately. Explanability refers primarily to the number of ways to communicate an ML solution to others (Hansen and Rieger, 2019), i.e., the “ways of exchanging information about a phenomenon, in this case the functionality of a model or the rationale and criteria for a decision, to different stakeholders.” Both properties of ML solutions are central to the broader adoption of such solutions in diverse high-stake healthcare scenarios, e.g., predicting the risk of complications to the health condition of a patient or the impact of treatment change.

While some authors (for instance, Hansen and Rieger, 2019; Mittelstadt et al., 2019; Samek and Müller, 2019) consider interpretability as an important component of explainability of ML solutions in AI, we view interpretability and explainability as complementary to each other, with interpretability being fundamental in ensuring trust in the results, transparency of the approach, confidence in deploying the results, and, where needed, quality of the maintenance of ML solutions. Further, in this study, we used the term interpretability in a broader sense, which subsumes communication and information exchange aspects of explainability.

We considered two connected aspects of the development of the overall concept of interpretability in ML solutions:

1. *methods*, which include the range of interpretable ML algorithms and interpretability solutions for AI/ML algorithms;
2. *methodologies* in data science, which consider explicitly the achievement of the necessary (for the project) interpretability of the ML solutions.

There is a wide collection of interpretable ML methods and methods for the interpretation of ML models. Murdoch et al. (2019) provide a compact and systematic approach toward their categorization and evaluation. Methods are categorized into model-based and *post-hoc* interpretation methods. They are evaluated using predictive accuracy, descriptive accuracy, and relevancy, the PDR framework (Murdoch et al., 2019), where relevancy is evaluated against human audience. The framework also provides common terminology for practitioners. Guidotti et al. (2018) and Carvalho et al. (2019) provide extensive systematic overviews with elaborate frameworks of the state-of-the-art of interpretability methods. Mi et al. (2020) provide broader taxonomy and comparative experiments, which can help practitioners in selecting suitable models with complementary features for addressing interpretability problems in ML solutions.

Model interpretability and explainability are crucial for clinical and healthcare practice, especially, since not only non-linear models but also inherently more interpretable ones, like decision trees, if large and complex, become difficult to comprehend (Ahmad et al., 2018).

On the other hand, working with data in the healthcare domain is complex at every step, starting from establishing and finding the relevant, typically numerous, diverse, and heterogeneous data sources required to address the research objective; integrating and mapping these data sources; identifying and resolving data quality issues; pre-processing and feature engineering without losing information or distorting it; and finally using the resulting high-dimensional, complex, sometimes unstructured, data to build a high-performing interpretable model. This complexity further supports the argument for the development of ML methodologies which explicitly embed interpretability through the data science project life cycle and ensure the achievement of the level of interpretability of ML solutions that had been agreed for the project. Interpretability of an ML solution can serve a variety of stakeholders involved in data science projects in connection with the implementation of their outcomes.

Interpretability of an ML solution can serve a variety of stakeholders, involved in data science projects and related to the implementation of their outcomes in algorithmic decision making (Berendt and Preibusch, 2017). For instance, the human-centric visual analytics methodology “Extract-Explain-Generate” for interrogating biomedical data (Kennedy et al., 2008) explicitly relates different stakeholders (molecular biologist, clinician, analysts, and managers) with specific areas of knowledge extraction and understanding associated with the management of patients. This study is focused on addressing the methodological challenges and opportunities of broad embedding of interpretability (including the selection of methods of interpretability that are appropriate for a project, given its objectives and constraints).

## 2. Challenges and Opportunities in Creating Methodologies Which Consistently Embed Interpretability

In order to progress with the adoption of ML in healthcare, a consistent and comprehensive methodology is needed: first, to minimize the risk of project failures, and second, to establish and ensure the needed level of interpretability of the ML solution while addressing the above-discussed diverse requirements to ML solutions. The rationale supporting these needs is built on a broader set of arguments about:

– the high proportion of data science project failures, including those in healthcare;
– the need to support an agreed level of interpretability and explainability of ML solutions;
– the need for consistent measurement and evaluation of interpretability of ML solutions; and
– the emerging need for standard methodology, which explicitly embeds mechanisms to manage the achievement of the level of interpretability of ML solutions required by stakeholders through the project.

Further, in this section, we use these arguments as dimensions around which we elaborate the challenges and opportunities for the design of cross-industry data science methodology, which is capable of handling interpretability of ML solutions under the complexity of the healthcare domain.

### 2.1. High Proportion of Data Science Project Failures

Recent reports, which include healthcare-related organizations, estimate that up to 85% of data science/ML/AI projects do not achieve their stated goals. The latest NewVantage Partners Big Data and AI Executive Survey, based on the responses from C-Executives from 85 blue-chip companies of which 22% are from Healthcare and Life Sciences, noted that only 39% of companies are managing data as an asset (NewVantage Partners LLC, 2021). Fujimaki (2020) emphasized that “the economic downturn caused by the COVID-19 pandemic has placed increased pressure on data science and BI teams to deliver more with less. In this type of environment, AI/ML project failure is simply not acceptable.” On the other hand, the NewVantage Partners survey (NewVantage Partners LLC, 2021) emphasized that, over the 10 years of conducting these surveys, organizations continue to struggle with their transformation into data-driven organizations, with only 29% achieving transformational business outcomes. Only 24% have created a data-driven organization, a decline from 37.8%, and only 24% have forged a data culture (NewVantage Partners LLC, 2021), a result which, to a certain extent, is counterintuitive to the overall expectation of the impact of AI technologies to decision-making and which projected benefits from the adoption of such technologies.

A number of sources (e.g., vander Meulen and Thomas, 2018; Kaggle, 2020; NewVantage Partners LLC, 2021) established that a key reason for these failures is linked to the lack of proper process and methodology in areas, such as requirement gathering, realistic project timeline establishment, task coordination, communication, and designing a suitable project management framework (see also Goodwin, 2011; Stieglitz, 2012; Espinosa and Armour, 2016). Earlier works have suggested (see, e.g., Saltz, 2015) that improved methodologies are needed as the existing ones do not cover many important aspects and tasks, including those related to interpretability (Mariscal et al., 2010). Further, studies have shown that the biased focus on the tools and systems has limited the ability to gain value from the effort of organizational analytics effort (Ransbotham et al., 2015) and that data science projects need to increase their focus on process and task coordination (Grady et al., 2014; Gao et al., 2015; Espinosa and Armour, 2016). A recent Gartner Consulting report also emphasizes the role of processes and methodology (Chandler and Oestreich, 2015) and practitioners agree with this view (for examples and analyses from diverse practical perspectives see Goodson, 2016; Arcidiacono, 2017; Roberts, 2017; Violino, 2017; Jain, 2019).

### 2.2. Support for the Required Level of Interpretability and Explainability of ML Solutions

In parallel with the above-discussed tendencies, there is pressure on the creation of frameworks/methodologies, which can ensure the necessary interpretability for sufficient explainability of the output of the ML solutions. While it has been suggested, in recent years, that it is only a matter of time before ML will be universally used in healthcare, building ML solutions in the health domain proves to be challenging (Ahmad et al., 2018). On the one hand, the demands for explainability, model fidelity, and performance in general in healthcare are much higher than in most other domains (Ahmad et al., 2018). In order to build the trust in ML solutions and incorporate them in routine clinical and healthcare practice, medical professionals need to clearly understand how and why an ML solution-driven decision has been made (Holzinger et al., 2017; Vellido, 2020).

This is further affected by the fact that the ML algorithms that achieve a high level of predictive performance, e.g., boosted trees (Chen and Guestrin, 2016) or deep neural networks (Goodfellow et al., 2016), are quite complex and usually difficult to interpret. In fact, some researchers argue that performance and interpretability of an algorithm are in reverse dependence (Ahmad et al., 2018; Molnar et al., 2019). Additionally, while there are a number of techniques aiming to explain the output of the models that are not directly interpretable, as many authors note (e.g., Holzinger et al., 2017; Gilpin et al., 2019; Rudin, 2019; Gosiewska et al., 2020), current explanatory approaches, while promising, do not seem to be sufficiently mature. Molnar et al. (2019) found that the reliability of some of these methods deteriorates if the number of features is large or if the level of feature interactions is high, which is often the case in health data. Further, Gosiewska and Biecek (2020) showed that current popular methods for explaining the output of ML models, like SHAP (Lundberg and Lee, 2017) and LIME (Ribeiro et al., 2016), produce inconsistent results, while Alvarez-Melis and Jaakkola (2018) found that the currently popular interpretability frameworks, particularly model-agnostic perturbation-based methods, are often not robust to small changes of the input, which clearly is not acceptable in the health domain.

There is a firm recognition of the impact of ML solutions in economics, including health economics, especially in addressing “predictive policy” problems (Athey, 2019). Many authors (e.g., Holzinger et al., 2017; Dawson et al., 2019; Rudin, 2019) note that in the high-stake areas (e.g., medical field, healthcare) solutions, in which the inner workings are not transparent (Weller, 2019), can be unfair, unreliable, inaccurate, and even harmful. Such views are reflected in the legislation on data-driven algorithmic decision-making, which affects citizens across the world. The European Union's General Data Protection Regulation (GDPR) (EU, 2016), which entered into force in May 2018, is an example of such early legislation. In the context of the emerging algorithmic economy, there are also warnings to policymakers to be aware of the potential impact of legislations like GDPR on the development of new AI and ML solutions (Wallace and Castro, 2018).

These developments increased the pressure on creation of frameworks and methodologies, which can ensure sufficient interpretability of ML solutions. In healthcare, such pressure is amplified by the nature of the interactive processes, wherein neither humans nor the algorithms operate with unbiased data (Sun et al., 2020).

Major technology developers, including Google, IBM, and Microsoft, recommend responsible interpretability practices (see, e.g., Google, 2019), including the development of common design principles for human-interpretable machine learning solutions (Lage et al., 2019).

### 2.3. Consistent Measurement and Evaluation of Interpretability of ML Solutions

While there are a number of suggested approaches to measuring interpretability (Molnar et al., 2019), a consensus on the ways of measuring or evaluating the level of interpretability has not been reached. For example, Gilpin et al. (2019) found that the best type of explanation metrics is not clear. Murdoch et al. (2019) mentioned that, currently, there is confusion about the interpretability notion and a lack of clarity about how the proposed interpretation approaches can be evaluated and compared against each other and how to choose a suitable interpretation method for a given issue and audience. The PDR framework (Murdoch et al., 2019), mentioned earlier, is a step in the direction of developing consistent evaluations. Murdoch et al. (2019) further note that there is limited guidance on how interpretability can actually be used in data science life cycles.

### 2.4. The Emerging Need for Standard Methodology for Handling Interpretability

Having a good methodology is important for the success of a data science project. To our knowledge, there is no formal standard for methodology in the data science projects (see Saltz and Shamshurin, 2016). Through the years, the CRISP-DM methodology (Shearer, 2000) created in the late 1990s has become a de-facto standard, as evidenced from a range of works (see, e.g., Huang et al., 2014; Niño et al., 2015; Fahmy et al., 2017; Pradeep and Kallimani, 2017; Abasova et al., 2018; Ahmed et al., 2018). An important factor of its success is the fact that it is industry, tool, and application agnostic (Mariscal et al., 2010). However, the research community has emphasized that, since its creation, CRISP-DM had not been updated to reflect the evolution of the data science process needs (Mariscal et al., 2010; Ahmed et al., 2018). While various extensions and refined versions of the methodology, including IBM's Analytics Solutions Unified Method for Data Mining (ASUM-DM) and Microsoft's Team Data Science Process (TDSP), were proposed to compensate the weaknesses of CRISP-DM, at this stage, none of them has become the standard. In the more recent years, variations of CRISP-DM tailored for the healthcare (Catley et al., 2009) and medical domain, such as CRISP-MED-DM (Niaksu, 2015), have been suggested. The majority of organisations that apply a data analysis methodology prefers extensions of CRISP-DM (Schäfer et al., 2018). Such extensions are fragmented and either propose additional elements into the data analysis process, or focus on organisational aspects without the necessary integration of domain-related factors (Plotnikova, 2018). These might be the reasons for the observed decline of its usage as reported in studies by Piatetsky-Shapiro (2014), Bhardwaj et al. (2015), and Saltz and Shamshurin (2016). Finally, while methodologies from related fields, like the agile approach used in software development, are being considered for use in data science projects, there is no clear clarity on whether they are fully suitable for the purpose, as indicated by Larson and Chang (2016); therefore, we did not include them in the current scope.

This overall lack of consensus has provided an opportunity to reflect on the philosophy of the CRISP-DM methodology and create a comprehensive data science methodology, through which interpretability is embedded consistently into an ML solution. Such methodology faces a list of requirements:

– It has to take into account the different perspectives and aspects of interpretability, including model and process explainability and interpretability;
– It has to consider the desiderata of explainable AI (fidelity, understandability, sufficiency, low construction overhead, and efficiency) as summarized in Hansen and Rieger (2019);
– It needs to support consistent interaction of local and global interpretability of ML solutions with other established key factors in data science projects, including predictive accuracy, bias, noise, sensitivity, faithfulness, and domain specifics;

In addition, healthcare researchers have indicated that the choice of interpretable models depends on the use case (Ahmad et al., 2018).

In order to standardize the expectations for interpretability, some of these requirements have been addressed in the recently proposed CRISP-ML methodology (Kolyshkina and Simoff, 2019). In section 3, we will briefly discuss the major concepts differentiating CRISP-ML methodology. The CRISP-ML approach includes the concepts of *necessary level of interpretability* (NLI) and *interpretability matrix* (IM), described in detail by Kolyshkina and Simoff (2019), and therefore aligns well with the view of health researchers that the choice of interpretable models depends upon the application and use case for which explanations are required (Ahmad et al., 2018). To illustrate that, in section 4, we present a use case in the public health field that illustrates the typical challenges met and the ways CRISP-ML helped to address and resolve them.

## 3. CRISP-ML Methodology—Toward Interpretability-Centric Creation of ML Solutions

**Figure 1:**
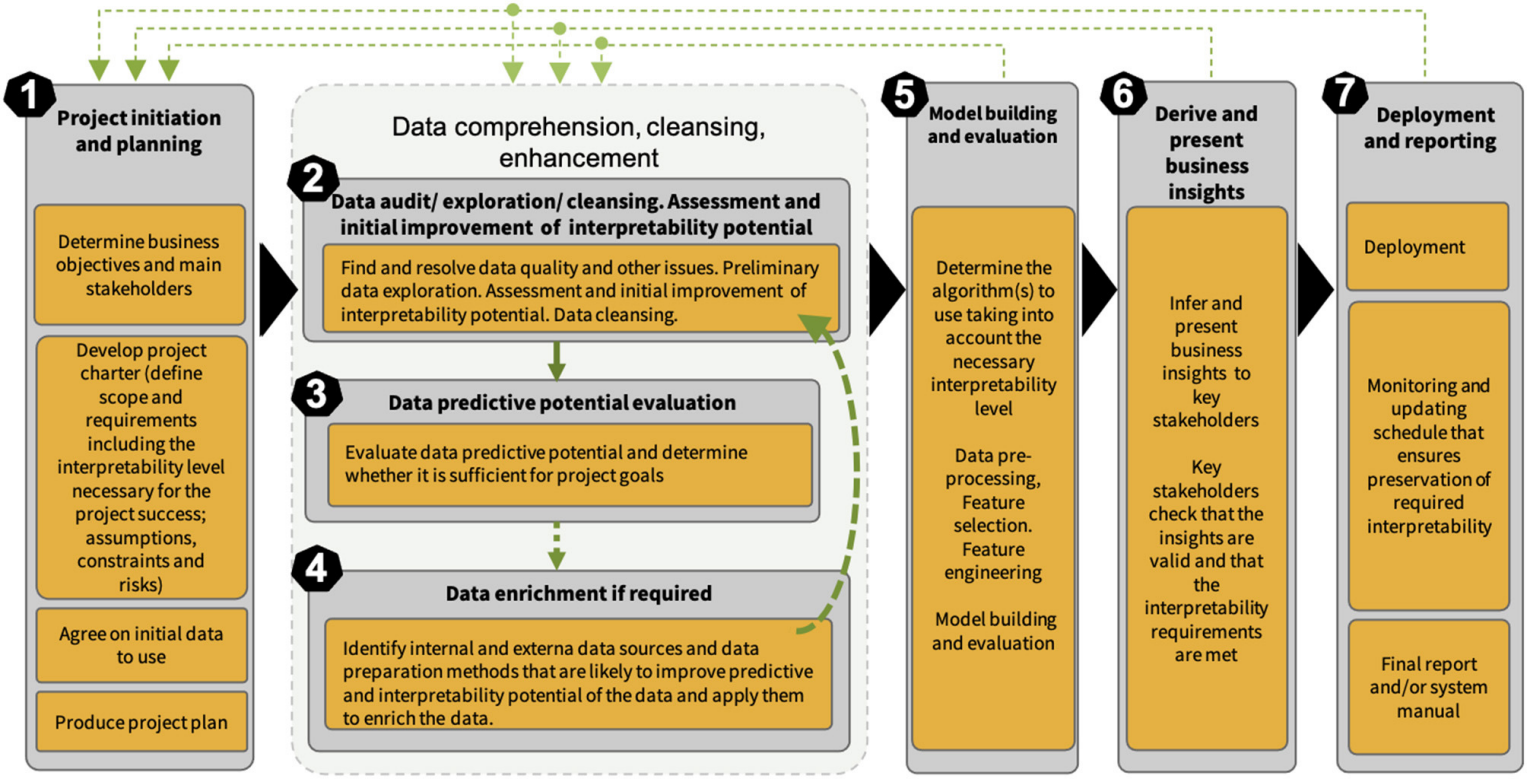
Conceptual framework of CRISP-ML methodology.

The CRISP-ML methodology (Kolyshkina and Simoff, 2019) of building interpretability of an ML solution is based on revision and update of CRISP-DM to address the opportunities discussed in section 2. It follows the CRISP-DM approach in terms of being industry-, tool-, and application-neutral. CRISP-ML accommodates the necessary elements to work with diverse ML techniques and create the right level of interpretability through the whole ML solution creation process. Its seven stages are described in Figure 1), which is an updated version of the CRISP-ML methodology diagram in the study by Kolyshkina and Simoff (2019).

Central to CRISP-ML is the concept of necessary level of interpretability of an ML solution. From this view point, CRISP-ML can be differentiated as a methodology of establishing and building the necessary level of interpretability of a business ML solution. In line with Google's guidelines on the responsible AI practices in the interpretability area (Google, 2019) and expanding on the approach proposed by Gleicher (2016), we have specified the concept of minimal necessary level of interpretability of a business ML solution as the combination of the degree of accuracy of the underlying algorithm and the extent of understanding the inputs, inner workings, the outputs, the user interface, and the deployment aspects of the solution, which is required to achieve the project goals. If this level is not achieved, the solution will be inadequate for the purpose. This level needs to be established and documented at the initiation stage of the project as part of requirement collection (see Stage 1 in Figure 1).

We then describe an ML solution as sufficiently interpretable or not based on whether or not it achieved the required level of interpretability. Obviously, this level will differ from one project to another depending on the business goals. If individuals are directly and strongly affected by the solution-driven decision, e.g., in medical diagnostics or legal settings, then both the ability to understand and trust the internal logic of the model, as well as the ability of the solution to explain individual predictions, are of highest priority. In other cases, when an ML solution is used in order to inform business decisions about policy, strategy, or interventions aimed to improve the business outcome of interest, then it is necessary to understand and trust the internal logic of the model that is of most value, while individual predictions are not the focus of the stakeholders. For example, in one of our projects, an Australian state organization wished to establish what factors influenced the proportion of children with developmental issues and what interventions can be undertaken in specific areas of the state in order to reduce that proportion. The historical, socioeconomic, and geographic data provided for the project was aggregated at a geographic level of high granularity.

In other cases, e.g., in the case of an online purchase recommender solution, the overall outcome, such as increase in sales volume, may be of higher importance than interpretability of the model. Similar requirements of solution interpretability were in a project where an organization owned assets that were located in remote areas and were often damaged by birds or animals nests. The organization wished to lower their maintenance cost and planning by identifying as soon as possible the assets where such nests were present instead of doing expensive examination of each asset. This was achieved by building a ML solution that classified Google Earth images of the assets into those with and without nests. In this project, it was important to identify a proportion of assets that were as high as possible with nests on them, while misclassifying an individual asset image was not of great concern.

The recently published CRISP-ML(Q) (Studer et al., 2020) proposes an incremental extension of CRISP-DM with the monitoring and maintenance phases. While the study mentions “model explainability” referring to the technical aspects of the underlying model, it does not consider interpretability and explainability in a systematic way as CRISP-ML (Kolyshkina and Simoff, 2019). Interpretability is now one of the most important and quickly developing universal requirements, not only a “best practice” requirement in some industries. It is also a legal requirement. CRISP-ML (Kolyshkina and Simoff, 2019) ensures that the necessary interpretability level is identified at the requirement collection stage. The methodology then ensures that participants establish the activities for each stakeholder group at each process stage that are required to achieve this level. CRISP-ML (Kolyshkina and Simoff, 2019) includes stages 3 and 4 (data predictive potential assessment and data enrichment in Figure 1), which are not present in CRISP-ML(Q) (Studer et al., 2020). As indicated in Kolyshkina and Simoff (2019), skipping these important phases can result in potential scope creep and even business project failure.

In Kolyshkina and Simoff (2019), the individual stages of the CRISP-ML methodology were presented in detail. Each stage was illustrated with examples from cases from a diverse range of domains. There, the emphasis was on the versatility of CRISP-ML as a industry-neutral methodology, including its approach to interpretability. In this study, we focus on a single case study from health-related domain in order to present a comprehensive coverage of each stage and the connections between the stages, and provide examples of how the required level of interpretability of the solution is achieved through carefully crafted involvement of the stakeholders as well as decisions made at each stage. This study does not provide comparative evaluation of CRISP-ML methodology in comparison to CRISP-DM (Shearer, 2000), ASUM-DM (IBM Analytics, 2015), TDSP (Microsoft, 2020), and other methodologies discussed by Kolyshkina and Simoff (2019). The purpose of the study is to demonstrate, in a robust way, the mechanics of explicit management of interpretability in ML through the project structure and life cycle of a data science methodology. Broader comparative evaluation of the methodology is the subject of a separate study.

The structure of the CRISP-ML process methodology has embedded flexibility in it, indicated by the cycles, which link the model-centric stages back to the early data-centric stages, as shown in Figure 1. Changes inevitably occur in any project over the course of the project life cycle, and CRISP-ML reflects that. The most typical changes, related to data availability, quality, and analysis findings, occur mostly at stages 2–4, as shown in Figure 1. This is illustrated in our case study and was discussed in detail in the study by Kolyshkina and Simoff (2019). Less often changes occur at stages 5–7 in Figure 1. From experiential observations, such changes are more likely to occur in longer projects with a volume of work requiring more than 6–8 months for completion. They are usually driven by amendments in project scope and requirements including the necessary level of interpretability (NLI), that are caused by factors external to the analytical part of the project. These factors can be global, such as environmental, political, or legislative factors; organization-specific (e.g., updates in the organizational IT structure, the way of data storage or changes in the stakeholder team), or they could be related to the progress in ML and ML-related technical areas (e.g., the advent of a new, better performing predictive algorithm).

In this study, we present the stages of CRISP-ML in a rigid manner, around the backbone of the CRISP-ML process, represented by the solid black triangle arrows in Figure 1 to maintain the emphasis on the mechanisms for handling interpretability in each of these steps, rather than exploring the iterative nature of the approach. For consistency of the demonstration, we draw all detailed examples through the study from the specific public health case study. As a result, we are able to illustrate in more depth how we sustain the level of interpretability through the process structure of the project. The study complements the study by Kolyshkina and Simoff (2019), where, through the examples drawn from a variety of cases, we demonstrated the versatility of CRISP-ML. The methodological treatment of interpretability in evolving scenarios and options is beyond the scope of this study.

## 4. Case Study Illustrating the Achievement of the NLI of Machine Learning Solution

In this study, we will describe a detailed real-world case study in which, by going through each project stage, we illustrate how CRISP-ML facilitates data science project stakeholders in establishing and achieving the necessary level of interpretability of ML solution.

We would like to emphasize that the specific analytic techniques and tools mentioned in the respective stages of the case study are relevant specifically to this particular study. They illustrate the approach and the content of the interpretability mechanisms of CRISP-ML. However, there are many other available methods and method combinations that can achieve the objectives of this and other projects.

We place a particular focus on the aspects and stages of CRISP-ML from the perspective of demonstrating the flow and impact of interpretability requirements and on how they have been translated into the necessary level of interpretability of the final ML solution. Further, the structure of this section follows the stages of CRISP-ML process structure in Figure 1. All sensitive data and information have been masked and altered to protect privacy and confidentiality, without loss of the sensible aspects relevant to this presentation.

### 4.1. Background. High-Level Project Objectives and Data Description

An Australian State Workers Compensation organization sought to predict, at an early stage of a claim, the likelihood of the claim becoming long-term, i.e., a worker staying on income support for 1 year or more from the date of lodgement. A further requirement was that the prediction model should be easily interpretable by the business.

The data that the analysis was to be based upon were identified by the organizational experts, based on the outcomes for about 20,000 claims incurred in the recent years, and included the following information:

– injured worker attributes, e.g., date of birth, gender, occupation, average weekly earnings, residential address;
– injury attributes, e.g., injury date, the information on the nature, location, mechanism, and agency of injury coded according to the National Type of Occurrence Classification System^2^;
– employer attributes (size, industry classification);
– details of all worker's income support or similar payments.

### 4.2. Building the Project Interpretability Matrix: An Overall Approach

Interpretability matrix is usually built at Stage 1 of the project as part of the requirement collection process. Data science practitioners recognize Stage 1 as crucial for the overall project success (see, e.g., PMI, 2017), as well as from the solution interpretability building perspective (Kolyshkina and Simoff, 2019).

The IM as a structure for capturing and translating interpretability requirements into specific actions and activities is generalized. However, the specific content of its cells depends on the project. Kolyshkina and Simoff (2019) demonstrated the CRISP-ML stages consistently applied to different projects across a number of industries, data sets, and data types.

It covers the activities needed to start up the data science project: (a) the identification of key stakeholders; (b) documenting project objectives and scope; (c) collecting requirements; (d) agreeing upon initial data; (e) preparing a detailed scope statement; and (f) developing project schedule and plan. The deliverable of this stage was a project charter documenting the above activities.

#### 4.2.1. Interpretability-Related Aspects of the Project Charter: Business Objectives, Main Stakeholders, and Interpretability Level

We will describe in more detail the aspects of the project charter that were directly related to this study, specifically the established business objectives, main stakeholders, and the established necessary interpretability requirements.

##### 4.2.1.1. *Business objectives and main stakeholders*.

The established objectives included:

1. Build an ML system that will explain what factors and to what extent influence the outcome, i.e., claim duration;
2. Allow the organization to derive business insights that will help make data-driven accurate decisions regarding what changes can be done to improve the outcome, i.e., reduce the likelihood of a long claim by a specified percentage;
3. Be accurate, robust, and work with real-world organizational data;
4. Have easy-to-understand outputs that would make sense to the executive team and end users (case managers) and that the end users could trust;
5. Present the output as business rules that are easy to understand for end users and to deploy, monitor, and update in organizational data.
6. Ensure that the overall ML solution is easy to understand and implement by the Information Technology (IT) team of the organization and to monitor/update the Business Intelligence (BI) team of the organization.

The *main stakeholders* were identified as follows: Executive team (E); End Users/Domain Experts, i.e., Case management team (DE); Information Technology team who would implement the solution in the organizational data (IT); Business Intelligence team who would monitor the solution performance and update the underlying model (BI); and Modeling team (M). These abbreviations are used further in the descriptions of the stages of the IM.

##### 4.2.1.2. *The established necessary interpretability level*.

The necessary interpretability level (Kolyshkina and Simoff, 2019) was established as follows.

– The E, IT, and DE teams needed to have a clear understanding of all internal and external data inputs to be used: their reliability, quality, and whether the internal inputs were representative of the organizational data that the solution would be deployed on.
– The E and DE teams needed to have a clear understanding of the high-level data processing approach (e.g., missing values treatment, aggregation level), as well as high-level modeling approach and its proven validity.
– The outputs needed to be provided in the form of easily understandable business rules. The E and DE teams needed to gain a clear understanding of the rules and to be able to assess their business validity and usefulness from the business point of view.
– The BI team, who would monitor the solution performance and update it as required, need to have a clear understanding of:
  – the data processing stage, as well as the modeling algorithm, its validity, and suitability from the ML point of view;
  – how to assess the solution performance and how the solution needs to be audited, monitored, and updated, as well as how often this should occur.
– The IT team, who would deploy the solution needed to have a clear understanding of the format of the output and confirm that it can be deployed in the organizational data within the existing constraints (e.g., resources, cost) and without disrupting the existing IT systems.

#### 4.2.2. Creating the Project IM: An Overall Approach

The next step is to create and fill out the IM, whose rows show CRISP-ML stages, and columns represent key stakeholders. In each cell of the matrix, we showed what needs to be done by each stakeholder at each project stage to ensure that the required level of solution interpretability is achieved. Matrix cells can be grouped horizontally when there are common requirements for a group of stakeholders. Matrix cells can be grouped vertically when there are common requirements for a specific stakeholder across a number of stages in CRISP-ML. This matrix, once completed, becomes part of the business requirements document. The activities it outlines are integrated into the project plan and are reviewed and updated along with the project plan.

##### 4.2.2.1. *Definition of stakeholder involvement extent*.

We define the extent of involvement of a stakeholder group needed to achieve the necessary interpretability level in a particular project stage as follows:

– high extent of involvement—the stakeholder group needs to be directly and actively involved in the solution development process to ensure that the NLI is achieved at the stage;
– medium extent of involvement—the stakeholder group needs to receive detailed regular updates on the progress of the stage and get directly involved in the work from time to time to ensure that the NLI is achieved at the stage. For example, this can refer to DE and IT providing information helping to better understand data sources and business processes of the organization.
– low extent of involvement—the stakeholder group is kept informed on the general progress of the stage.

**Figure 2:**
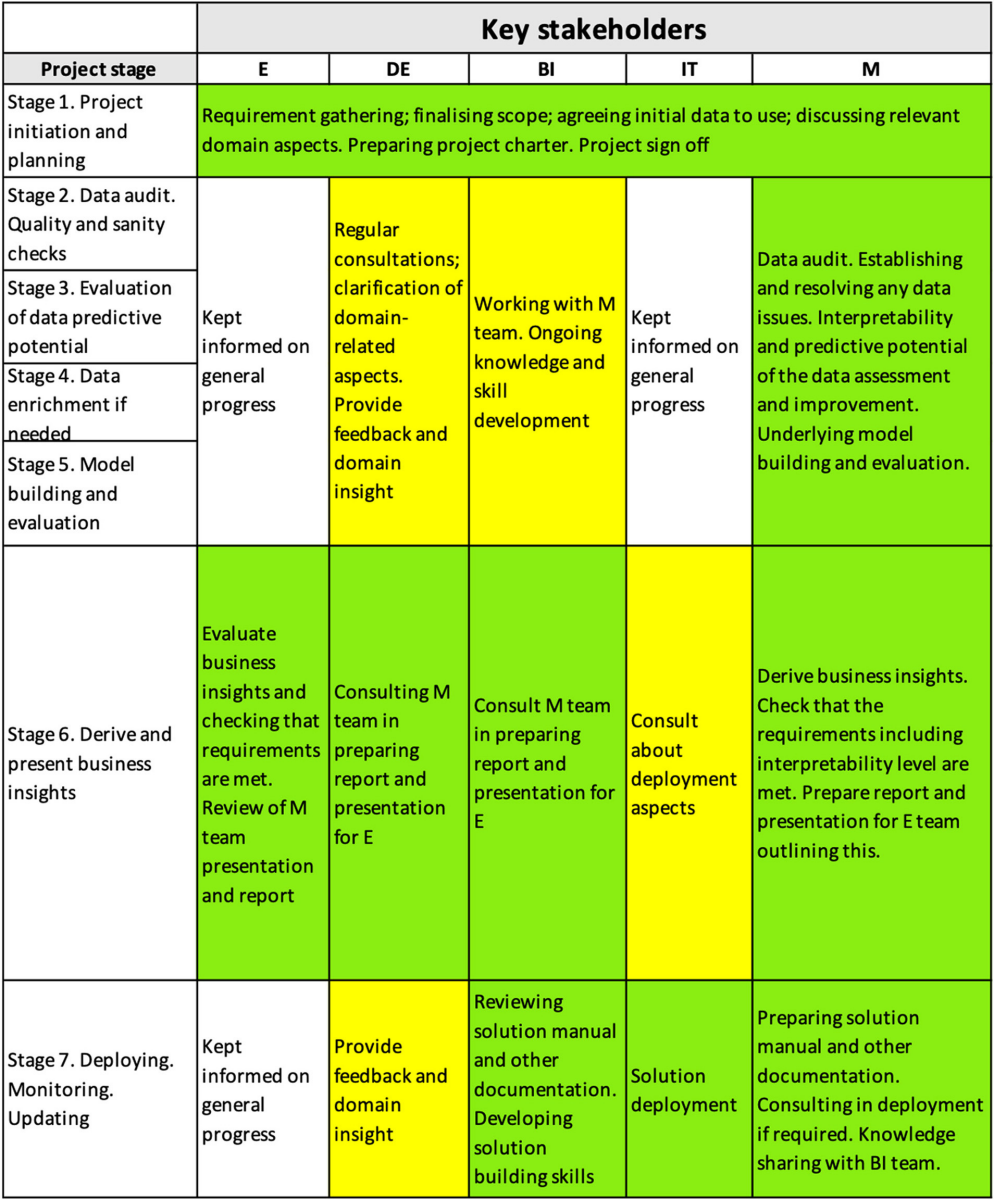
High-level interpretability matrix for the project.

In Figure 2, green color background indicates high extent of involvement of a stakeholder group, yellow color shows medium extent of involvement, and the cells with no color in the background show low level of involvement. Depending on the project, the coloring of the cells of the IM will vary. For example, if it had not been necessary to provide knowledge transfer (“Ongoing knowledge and skill development”) to the BI team, then their involvement in Stage 2–5 would have been low and the respective cells would have been left with no color in the background.

##### 4.2.2.2. *High-level IM diagram*.

Figure 2 shows a high-level interpretability matrix for the project.

### 4.3. Entries to the Project Interpretability Matrix at Each Stage of CRISP-ML

Further, we discuss entries to the project IM at each stage of CRISP-ML.

#### 4.3.1. Stage 1

**Figure 3:**
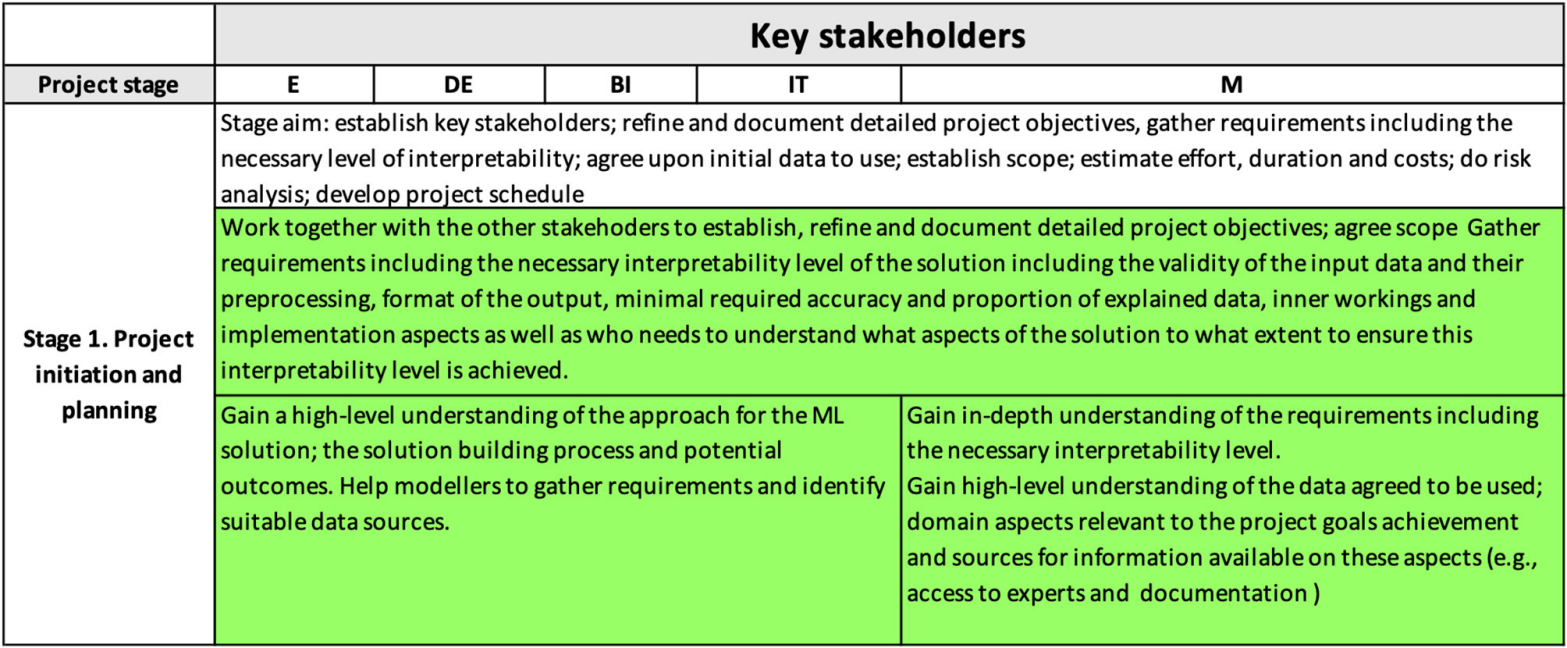
Interpretability matrix content for Stage 1.

The content of the interpretability matrix related to the project initiation and planning stage (i.e., the first row of the matrix) has been discussed in detail above and is summarized in Figure 3.

#### 4.3.2. Stages 2–4

Stages 2–4 in Figure 1 are mainly data-related and form the data comprehension, cleansing, and enhancement mega-stage. Further, we consider the content of the interpretability matrix for each of these stages, they are represented by the second, third, and fourth rows of interpretability matrix.

##### 4.3.2.1. *Stage 2*.

**Figure 4:**
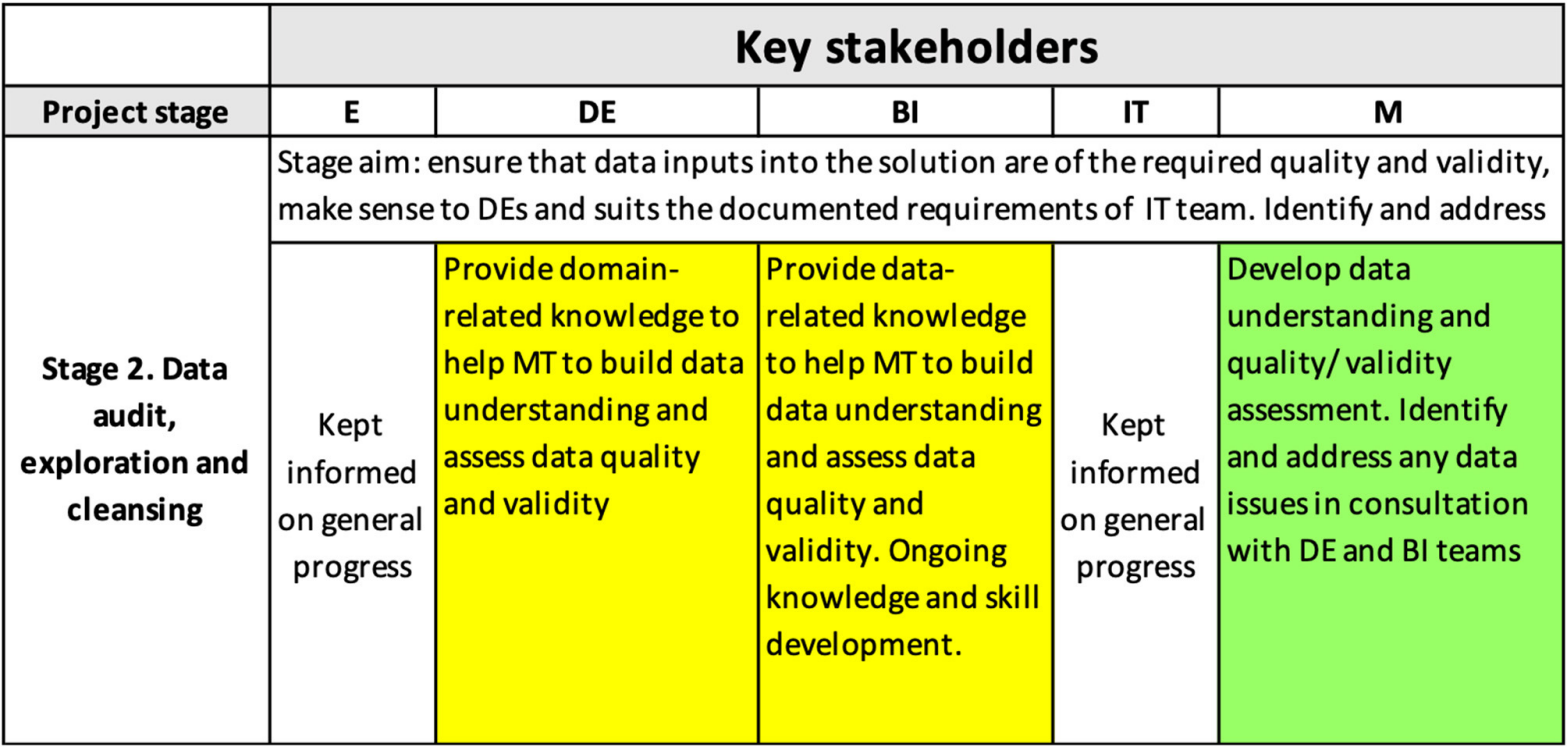
Interpretability matrix content for Stage 2.

Data audit, exploration, and cleansing played a key role in achieving the interpretability level needed for the project. Figure 4 demonstrates the content of the interpretability matrix at this stage.

This stage established that the data contained characteristics that significantly complicated the modeling, such as a large degree of random variation, multicollinearity, and a highly categorical nature of many potentially important predictors. These findings helped guide the selection of the modeling and data pre-processing approach.

*Random variation*. During workshops with E, DE, and other industry experts, it became clear that there were certain “truths” that pervaded the industry, and we used these to engage with subject matter experts (SME) and promote the value of our modeling project. One such “truth” was that claim duration was influenced principally by nature and location of injury, but in combination with the age of the injured worker, and specifically, older workers tended to have longer duration claims. Our analysis demonstrated the enormous amount of random variation that existed in the data. For example, age, body location, and injury type only explained 3–7% of variation in claim duration. There was agreement among the experts that the industry “truths” were insufficient to accurately triage claims and that different approaches were needed.

Our exploratory analysis revealed strong random variation in the data, confirming the prevalent view among the workers' compensation experts that it is the intangible factors, like the injured worker's mindset and relationship with the employer, that play the key role in the speed of recovery and returning to work. The challenge for the modeling, therefore, was to uncover the predictors that represent these intangibles.

*Sparseness*. Most of the available variables were categorical with large numbers of categories. For example, the variable “Injury Nature” has 143 categories and “Body Location of Injury” has 76 categories. Further, some categories had relatively few observations which made any analysis involving them potentially unreliable and not statistically valid. Such sparseness presented another data challenge.

*Multicollinearity*. There was a high degree of multicollinearity between numerical variables in the data.

*Data pre-processing*. First, we reduced the sparseness among categories by combining some categorical levels in consultation with SMEs to ensure that the changes made business sense. Second, we used a combination of correlation analysis, as well as advanced clustering and feature selection approaches, e.g., Random Forests (see, e.g., Shi and Horvath, 2006) and PCAMIX method using iterative relocation algorithm and ascendant hierarchical clustering (Chavent et al., 2012) to reduce multicollinearity and exclude any redundant variables.

##### 4.3.2.2. *Stage 3*.

**Figure 5:**
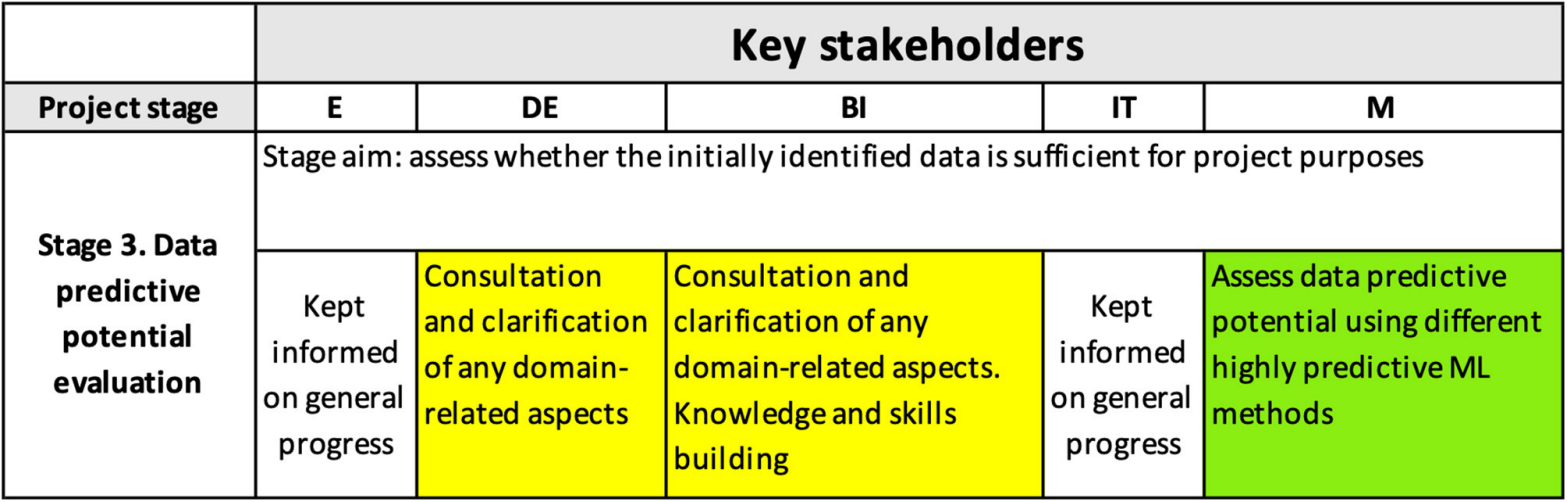
Interpretability matrix content for Stage 3.

Figure 5 shows the content of the interpretability matrix related to the evaluation of the predictive potential of the data (i.e., the third row of the matrix). This stage is often either omitted or not stated explicitly in other processes/frameworks (Kolyshkina and Simoff, 2019); however, it is crucial for the project success because it establishes whether the information in the data is sufficient for achieving the project goals.

To efficiently evaluate what accuracy could be achieved with the initially supplied data, we employed the following different data science methods that have proven their excellence at extracting maximum predictive power from the data: Deep Neural Nets, Random Forests, XGBoost, and Elastic Net. The results were consistent for all the methods used and showed that only a small proportion of the variability of claim duration was explained by the information available in the data. Therefore, the predictive potential of the initially supplied data, containing claim and worker's data history, indicated that the data set is insufficient for the project objectives. Data enrichment was required.

These findings were discussed with DE who then were invited to share their business knowledge about sources that could enrich the initial data predictive power.

##### 4.3.2.3. *Stage 4*.

**Figure 6:**
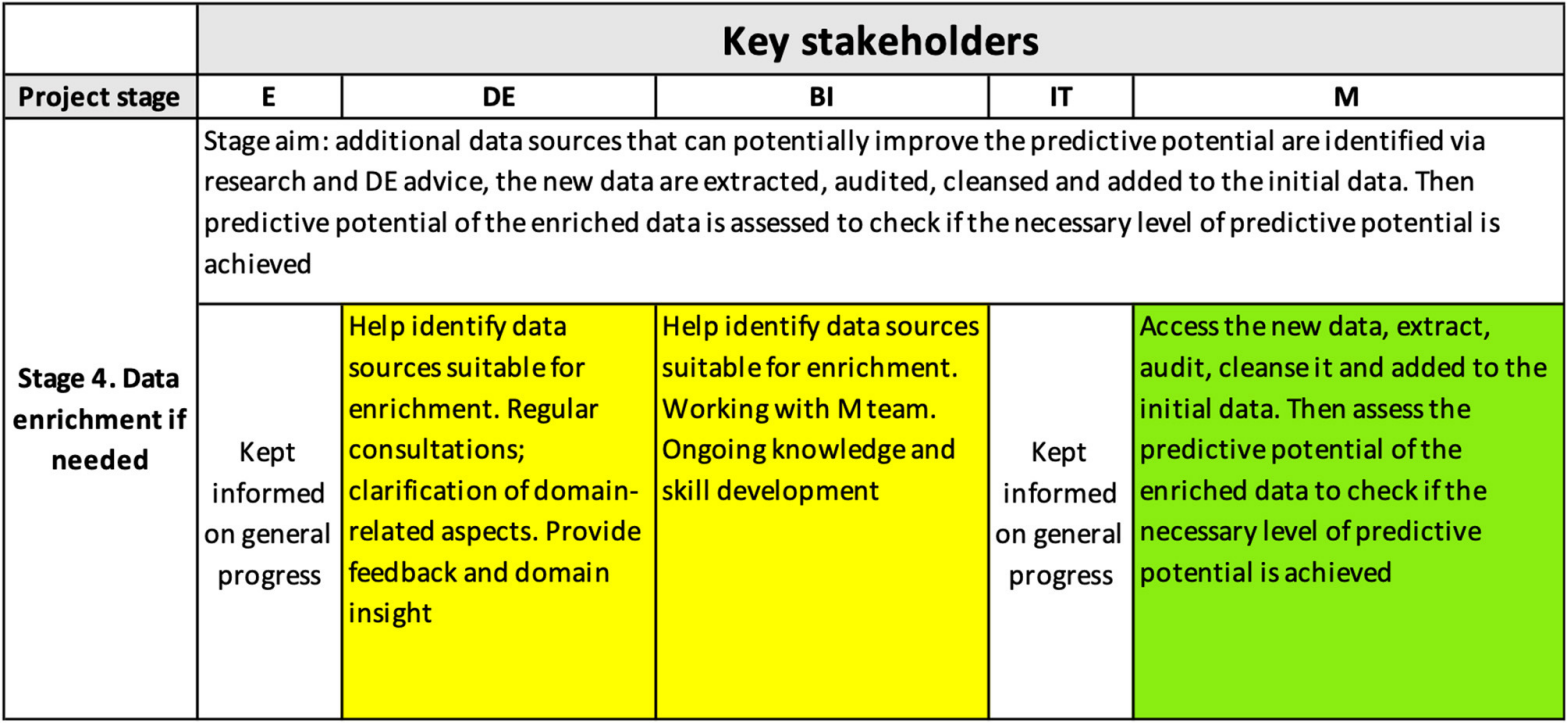
Interpretability matrix content for Stage 4.

*Data enrichment*. Figure 6 shows the content of the interpretability matrix related to the data enrichment stage. Based on the DE feedback and results of external research, we enriched the data with additional variables, including:

– lag between injury occurrence and claim lodgement (claim reporting lag);
– information on the treatment received (e.g., type of providers visited, number of visits, provider specialty);
– information on the use of medications and, specifically, on whether a potent opioid was used.

We assessed the predictive value of the enriched data in the same way as before (see section 4.3.2.2), and found that there was a significant increase in the proportion of variability explained by the model. Of particular relevance was the incorporation of the prior claim history of claimants, including previous claim count, type and nature of injury, and any similarity with the current injury.

Further, the data enrichment was a key step in building further trust of the DE team. The fact that the model showed that the cost of a claim can be significantly dependent on the providers a worker visited built further trust in the solution, because it confirmed the hunch of domain experts that they previously had not had enough evidence to prove.

#### 4.3.3. Stage 5

**Figure 7:**
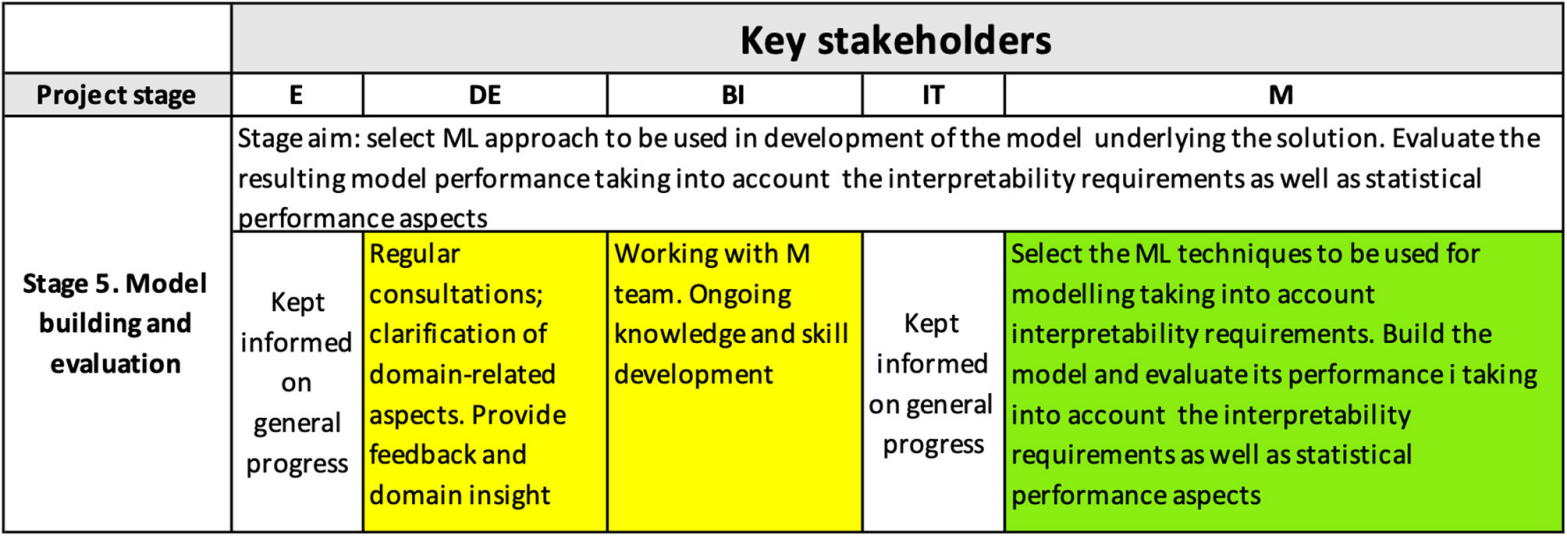
Interpretability matrix content for Stage 5.

Figure 7 shows the content of the interpretability matrix for the model building and evaluation stage. To achieve the right interpretability level, it is crucial that modelers choose the right technique that will balance the required outcome interpretability with the required level of accuracy of the model, which is often a challenge (see, e.g., Freitas, 2014), as well as with other requirements/constraints (e.g., the needed functional form of the algorithm). In our case, it was required that the model explained at least 70% of variability.

At this stage, the ML techniques to be used for modeling are selected, taking into account the predictive power of the model, its suitability for the domain and the task, and the NLI. The data is pre-processed, and modeled, and the model performance is evaluated. The solution output was required to be produced in the form of business rules, and therefore, the feature engineering methods and modeling algorithms used included rule-based techniques, e.g., decision trees, and association rules-based methods.

#### 4.3.4. Stage 6

**Figure 8:**
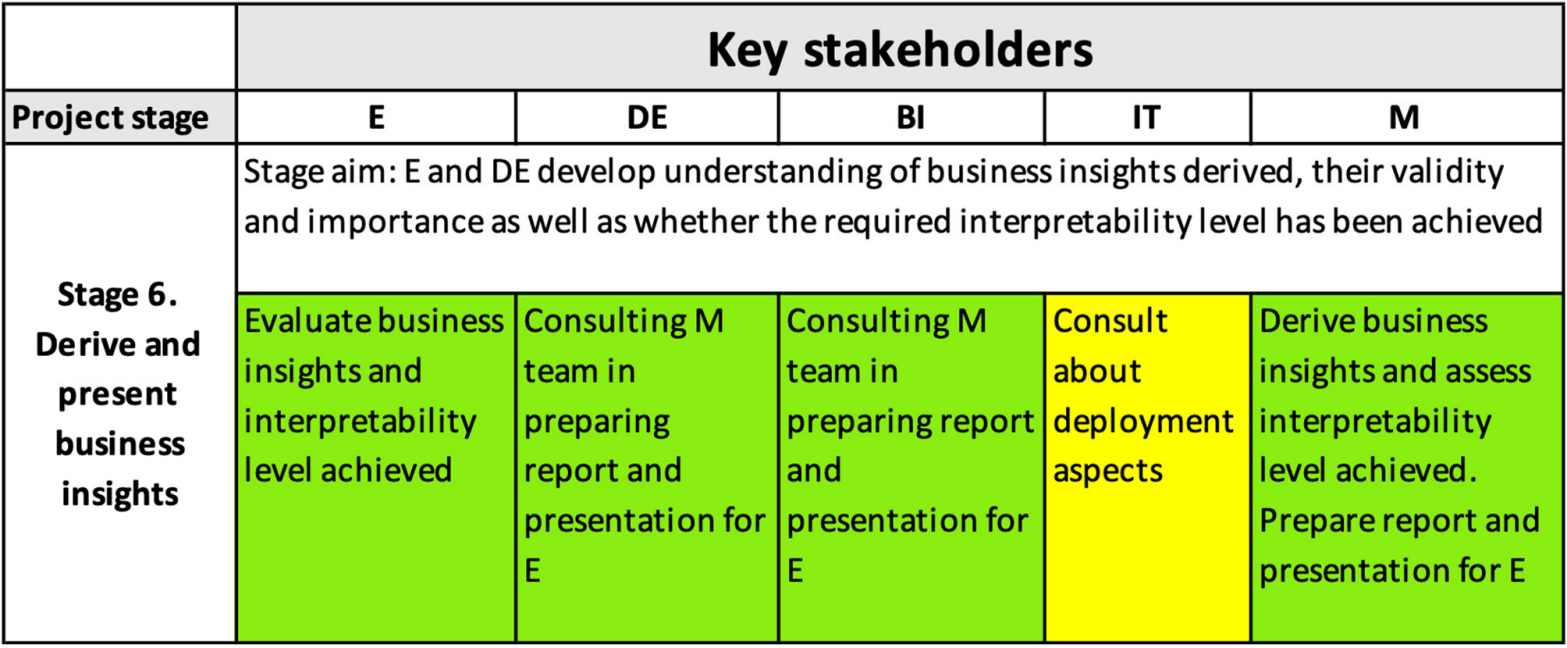
Interpretability matrix content for Stage 6.

Figure 8 shows how the interpretability matrix reflects the role of interpretability in the formulation of business insights necessary to achieve the project goals and in helping the E and DE to understand the derived business insights and to develop trust in them. Modelers, BI and DEs, prepared a detailed presentation for the E, explaining not only the learnings from the solution but also the high-level model structure and its accuracy.

#### 4.3.5. Stage 7

The final model provided the mechanism for the organization to allocate claims to risk segments based on the information known at early stages. From the technical point of view, the business rules were confirmed by the E, DE, and IT to be easy to deploy as they are readily expressed as SQL code. Based on this success, a modified version of claims triage was deployed into production.

**Figure 9:**
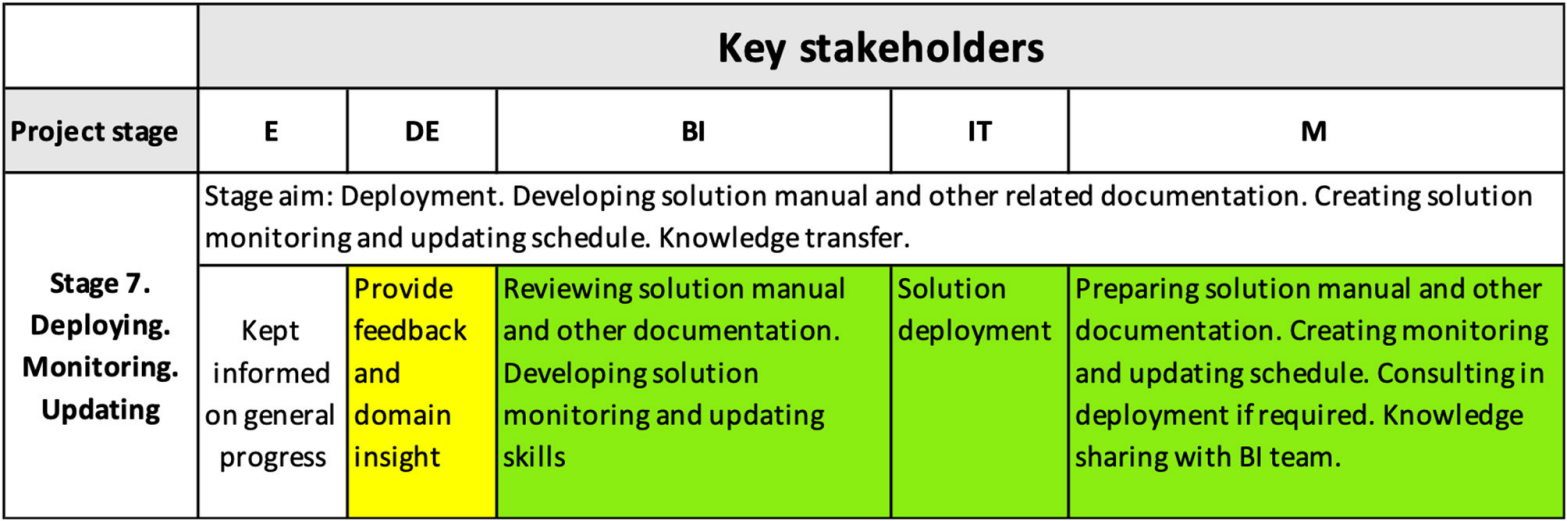
Interpretability matrix content for Stage 7 includes activities ensuring the achieved interpretability level is maintained during the future utilization of the solution.

Figure 9 shows the shift of responsibilities for ensuring the achieved interpretability level is maintained during the future use of the solution. At this stage, the deployment was being scheduled, and the monitoring/updating process and schedule was prepared, based on the technical report provided by the M team that included project code, the solution manual, and updating and monitoring recommendations.

## 5. Conclusions

This study contributes toward addressing the problem for providing organizations with capabilities to ensure that the ML solutions they develop to improve decision-making are transparent and easy to understand and interpret. If needed, the logic behind the decisions can be explained to any external party. Such capability is essential in many areas, especially in health-related fields. It allows the end users to confidently interpret the ML output use to make successful evidence-based decisions.

In an earlier study (Kolyshkina and Simoff, 2019), we introduced CRISP-ML, a methodology of determining the interpretability level required for the successful real-world solution and then achieving it *via* integration of the interpretability aspects into its overall framework instead of just the algorithm creation stage. CRISP-ML combines practical, common-sense approach with statistical rigor and enables organizations to establish shared understanding across all key stakeholders about the solution and its use and build trust in the solution outputs across all relevant parts of the organization. In this study, we illustrated CRISP-ML with a detailed case study of building an ML solution in the Public Health sector. An Australian state workplace insurer sought to use their data to establish clear business rules that would identify, at an earlier stage of a claim, those with high probability of becoming serious/long-term. We showed how the necessary level of solution interpretability was determined and achieved. First, we showed how it was established by working with the key stakeholders (Executive team, end users, IT team, etc.). Then, we described how the activities that were required to be included at each stage of building the ML solution to ensure that this level is achieved was determined. Finally, we described how these activities were integrated into each stage.

The study demonstrated how CRISP-ML addressed the problems with data diversity, unstructured nature of the data, and relatively low linkage between diverse data sets in the healthcare domain (Catley et al., 2009; Niaksu, 2015). The characteristics of the case study which we used are typical for healthcare data, and CRISP-ML managed to deliver on these issues, ensuring the required interpretability of the ML solutions in the project.

While we have not completed formal evaluation of CRISP-ML, there are two aspects which indicate that the use of this methodology improves the chances of success of data science projects. On the one hand, CRISP-ML is built on the strengths of CRISP-DM, which made it the preferred and effective methodology (Piatetsky-Shapiro, 2014; Saltz et al., 2017), addressing its identified limitations in previous works (e.g., Mariscal et al., 2010). On the other hand, CRISP-ML has been successfully deployed in a number of recent real-world projects across several industries and fields, including credit risk, insurance, utilities, and sport. It ensured on meeting the interpretability requirements of the organizations, regardless of industry specifics, regulatory requirements, types of stakeholders involved, project objectives, and data characteristics, such as type (structured as well as unstructured), size, or complexity level.

CRISP-ML is a living organism and, as such, it responds to the rapid progress in the development of ML algorithms and the evolution of the legislation for their adoption. Consequently, CRISP-ML development includes three directions: (i) the development of a richer set of quantitative measures of interpretability features for human interpretable machine learning, (ii) the development of the methodology and respective protocols for machine interpretation, and (iii) the development of formal process support. The first one is being extended in a way to provide input to the development and evaluation of common design principles for human interpretable ML solutions in line with that described in the study by Lage et al. (2019). This strategic development adds the necessary agility for the relevance of the presented cross-industry standard process.

## Data Availability Statement

The original contributions presented in the study are included in the article/supplementary material, further inquiries can be directed to the corresponding author/s.

## Author Contributions

All authors listed have made a substantial, direct and intellectual contribution to the work, and approved it for publication.

## Conflict of Interest

IK was employed by the company Analytikk Consulting Services. The remaining author declares that the research was conducted in the absence of any commercial or financial relationships that could be construed as a potential conflict of interest.

## Footnotes

^1^https://www.un.org/sustainabledevelopment/sustainable-development-goals/ and https://sdgs.un.org/goals.

^2^Type of Occurrence Classification System (3rd Edition, Revision 1), Australian Government—Australian Safety and Compensation Council, Canberra, 2008, https://www.safeworkaustralia.gov.au/doc/type-occurrence-classification-system-toocs-3rd-edition-may-2008).
